# Crystal structures of three *N*-ar­yl-2,2,2-tri­bromo­acetamides

**DOI:** 10.1107/S2056989015015248

**Published:** 2015-08-22

**Authors:** S. Sreenivasa, S. Naveen, N. K. Lokanath, G. M. Supriya, H. N. Lakshmikantha, P. A. Suchetan

**Affiliations:** a Department of Studies and Research in Chemistry, Tumkur University, Tumakuru, India; b Institution of Excellence, University of Mysore, Mysuru-6, India; c Department of Physics, University of Mysore, Mysuru-6, India; d University College of Science, Tumakuru, India; e Department of Chemistry, University College of Science, Tumkur University, Tumakuru 572013, India

**Keywords:** crystal structures, bromine⋯bromine contact, bromine⋯fluorine contact, *N*-ar­yl-tri­bromo­acetamides

## Abstract

Three *N*-ar­yl-2,2,2-tri­bromo­acetamides, namely, 2,2,2-tri­bromo-*N*-(2-fluoro­phen­yl)­acetamide, C_8_H_5_Br_3_FNO, (I), 2,2,2-tri­bromo-*N*-[3-(tri­fluoro­methyl)­phen­yl]­acetamide, C_9_H_5_Br_3_F_3_NO, (II) and 2,2,2-tri­bromo-*N*-(4-fluoro­phen­yl)­acetamide, C_8_H_5_Br_3_FNO, (III) were synthesized and their crystal structures were analysed. In the crystal structure of (I), C—Br⋯π_ar­yl_ inter­actions connect the mol­ecules into dimers, which in turn are connected *via* Br⋯Br contacts [3.6519 (12) Å], leading to the formation of a one-dimensional ladder-type architecture. The crystal structure of (II) features chains linked by N—H⋯O and C—H⋯O hydrogen bonds. Two such chains are inter­linked to form ribbons through Br⋯Br [3.6589 (1) Å] and Br⋯F [3.0290 (1) Å] inter­actions. C—Br⋯π_ar­yl_ and C—F⋯π_ar­yl_ inter­actions between the ribbons extend the supra­molecular architecture of (II) from one dimension to two. In (III), the mol­ecules are connected into *R*_2_^2^(8) dimers *via* pairs of C—H⋯F inter­actions and these dimers form ribbons through Br⋯Br [3.5253 (1) Å] contacts. The ribbons are further inter­linked into columns *via* C—Br⋯O=C contacts, forming a two-dimensional architecture.

## Abstract 2

Three *N*-(ar­yl)-2,2,2-tri­bromo­acetamides show different weak inter­actions in their crystal structures.

## Chemical context   

*N*-Ar­yl-halo­amides show a broad spectrum of pharmacological properties, including anti­bacterial (Manojkumar *et al.*, 2013*a* ▸), anti­tumor (Abdou *et al.*, 2004 ▸), anti-oxidant, analgesic and anti­viral activity (Manojkumar *et al.*, 2013*b* ▸). Keeping this in mind, and as a part of our ongoing efforts to understand the effect of the ring substituents on the mol­ecular and crystal structures of *N*-ar­yl-2,2,2-tri­bromo­acetamides Suchetan *et al.*, 2010 ▸) and also to study the role of different halogen inter­actions in solid-state structures, the crystal structures of three *N*-ar­yl-2,2,2-tri­bromo­acetamides, namely, 2,2,2-tri­bromo-*N*-(2-fluoro­phen­yl)­acetamide, (I), 2,2,2-tri­bromo-*N*-[3-(tri­fluoro­methyl)­phen­yl]­acetamide, (II) and 2,2,2-tri­bromo-*N*-(4-fluoro­phen­yl)­acetamide, (III), are discussed here.
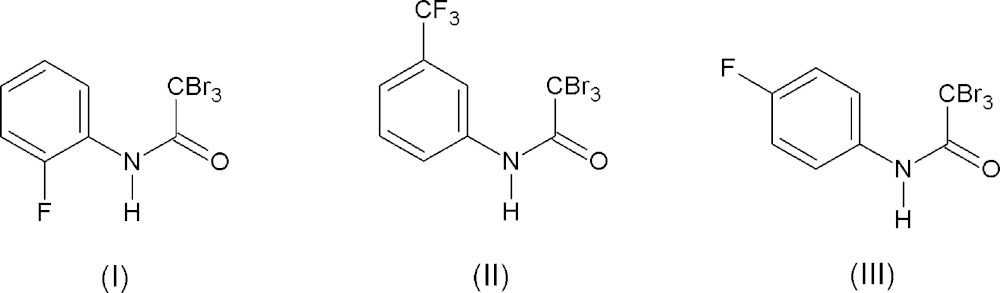


## Structural commentary   

**Figure 1:**
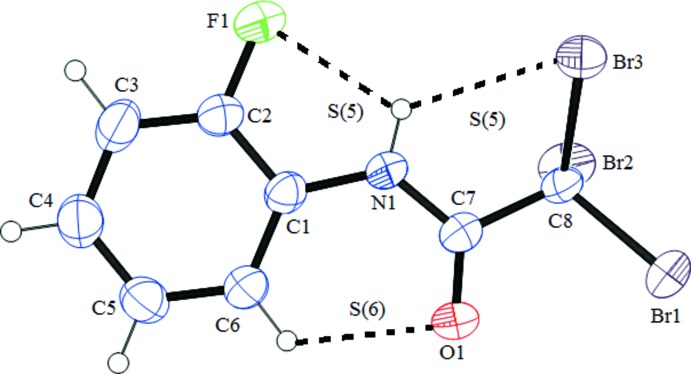
A view of (I), with displacement ellipsoids drawn at the 50% probability level.

The mol­ecular structures of (I), (II) and (III) are shown in Figs. 1 ▸, 2 ▸ and 3 ▸, respectively.

In (I), the conformation of the N—H bond is *syn* to the 2-fluoro substituent in the benzene ring, similar to that observed in the crystal structures of other *ortho* substituted compounds (see database survey). Contrast to the above, in (II), the conformation of the N—H bond is *anti* to the 3-CF_3_ substituent.

In (I), the dihedral angle between the benzene ring and the C1–N1–C7(O)–C8 segment is 4.2 (3)°, and, the various torsion angles defining the conformation between the benzene ring and the side chain have values closer to either 0 or 180°: C1—N1—C7—O1 = 0.2 (9), C1—N1—C7—C8 = 179.3 (5), C2—C1—N1—C7 = 175.8 (5) and C6—C1—N1—C7 = −4.0 (8)°. The mol­ecule (excluding three bromine atoms) is close to planar, the r.m.s. deviation (excluding H and Br atoms) being 0.031 (1) Å. The planarity is consolidated by three kinds of intra­molecular hydrogen bonds, namely, N1—H1⋯Br3, N1—H1⋯F1 and C6—H6⋯O1 (Fig. 1 ▸, Table 1 ▸).

**Figure 2:**
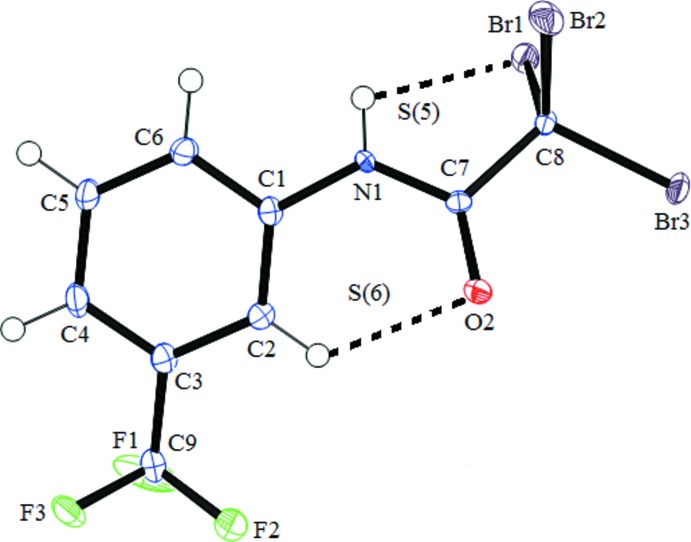
A view of (II), with displacement ellipsoids drawn at the 50% probability level.

The dihedral angle between the benzene ring and the C1–N1–C7(O)–C8 segment in (II) is 19.29 (1)°. The torsion angles are C1—N1—C7—O2 = −0.8 (7), C1—N1—C7—C8 = −177.3 (4), C2—C1—N1—C7 = −20.8 (7) and C6—C1—N1—C7 = 161.6 (4)°. These values deviate slightly from 0 or 180°, and thus mol­ecular planarity (excluding three bromine atoms) is not observed, the r.m.s. deviation (excluding H and Br atoms) being 0.159 (1) Å. The structure of (II) features two intra­molecular hydrogen bonds, namely, N1—H1⋯Br1 and C2—H2⋯O2 (Fig. 2 ▸, Table 2 ▸).

**Figure 3:**
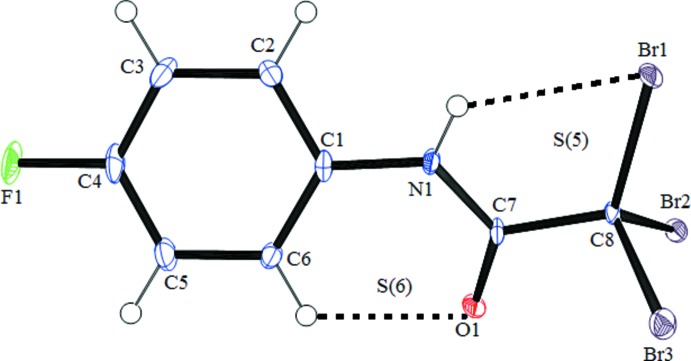
A view of (III), with displacement ellipsoids drawn at the 50% probability level.

The dihedral angle between the benzene ring and the C1–N1–C7(O)–C8 segment in (III) is highest among the three compounds, it being 22.5 (3)°. Similar to (II), the mol­ecular structure of (III) features two intra­molecular hydrogen bonds, namely, N1—H1⋯Br1 and C2—H2⋯O1 (Fig. 3 ▸, Table 3 ▸). Further, the various torsion angles defining the conformation between the benzene ring and the side chain show that the two are not in a single plane: C1—N1—C7—O1 = 4.2 (9), C1—N1—C7—C8 = −172.4 (5), C2—C1—N1—C7 = 19.8 (9) and C6—C1—N1—C7 = −164.0 (6)°.

## Supra­molecular features   

**Figure 4:**
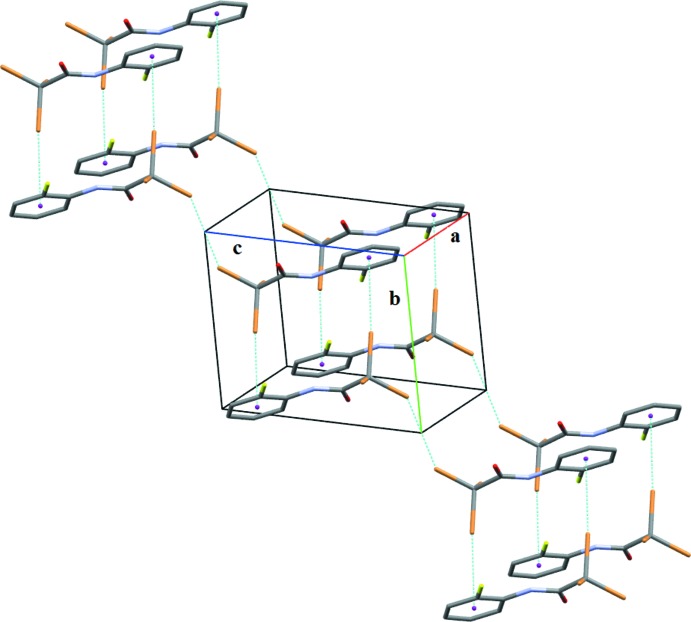
Crystal packing of (I), displaying C—Br⋯π and Br⋯Br contacts. H atoms are omitted for clarity.

In the crystal structure of (I), C8—Br2⋯π_ar­yl_ inter­actions (Table 4 ▸) connect the mol­ecules into dimers and these dimers are in turn connected via Br1⋯Br1 contacts [3.6519 (12) Å] along the diagonal of the *bc* plane, leading to the formation of a one-dimensional ladder-type architecture (Fig. 4 ▸, Table 4 ▸). The Br1⋯Br1 contact has a type I *trans* geometry (Dikundwar *et al.*, 2012 ▸) with θ_1_ = θ_2_ = 141.04 (14)°. The crystal structure of (I) does not feature the strong N—H⋯O hydrogen bonds which are generally observed in amides.

**Figure 5:**
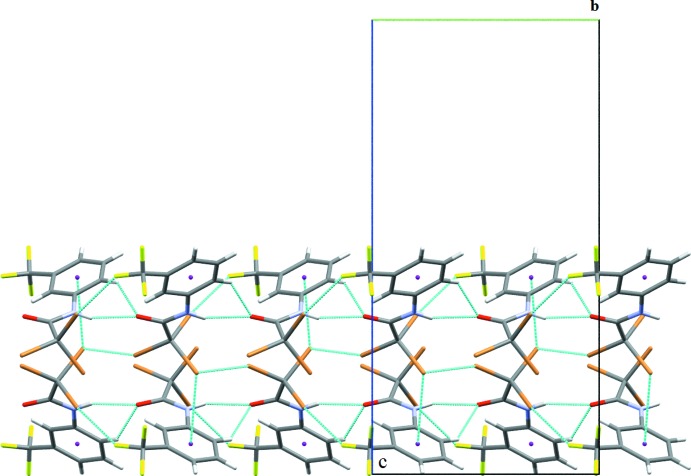
Crystal packing of (II), displaying various inter­actions of the types N—H⋯O, C—H⋯O, C—Br⋯π and Br⋯Br.

**Figure 6:**
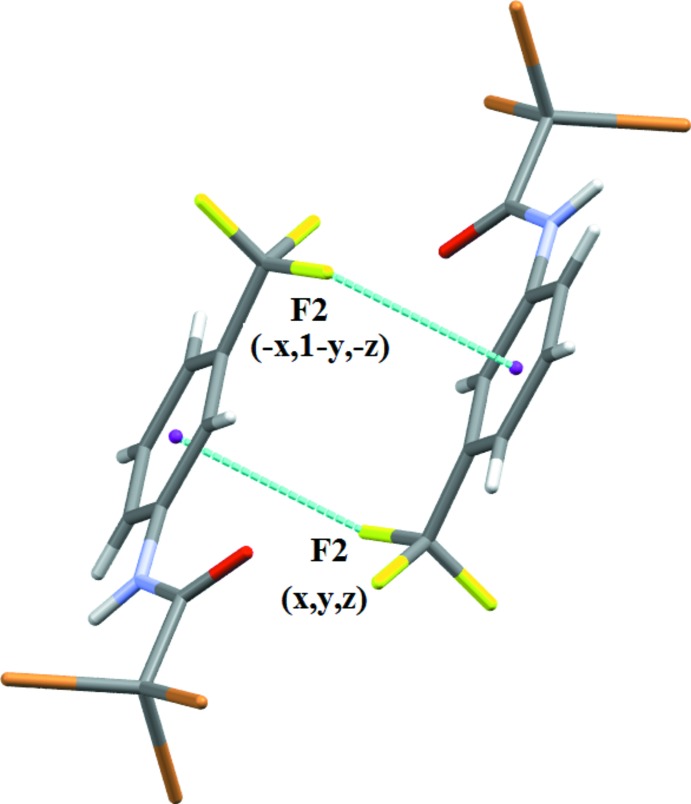
Crystal packing of (II), displaying C—F⋯π inter­actions.

The crystal structure of (II) features mol­ecular chains along [010] formed by N1—H1⋯O2 and C6—H6⋯O2 hydrogen bonds (Fig. 5 ▸ and Table 2 ▸). Two such chains are inter­linked to form ribbons through Br1⋯Br3 [3.6589 (1) Å] and Br2⋯F2 [3.0290 (1) Å] inter­actions (Fig. 6 ▸, Table 5 ▸). C8—Br1⋯π_ar­yl_ and C9—F2⋯π_ar­yl_ inter­actions between the ribbons extend the supra­molecular architecture of (II) from one dimension to two (Fig. 6 ▸, Table 5 ▸). The Br⋯Br contact in (II) is close to a type II halogen⋯halogen contact (Dikundwar *et al.*, 2012 ▸), while, Br⋯F is a type I *cis* contact.

**Figure 7:**
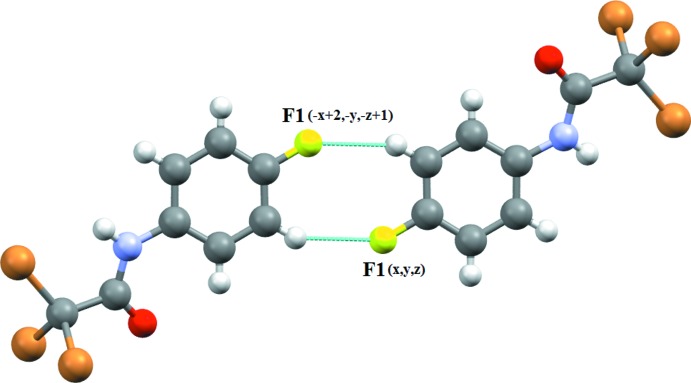
Formation of 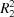
(8) dimers *via* C—H⋯F inter­actions in (III).

**Figure 8:**
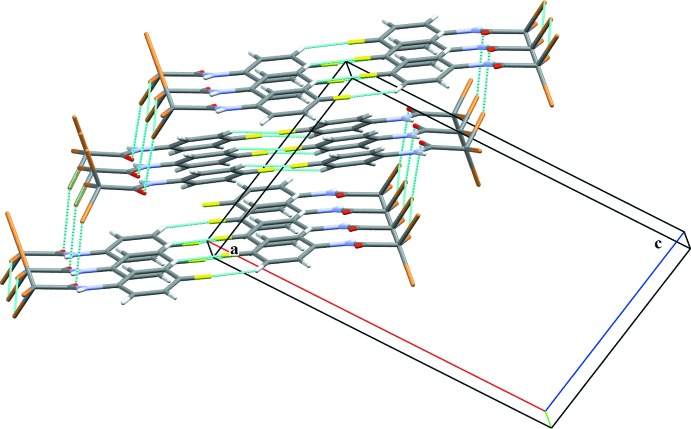
Column-like architecture displayed in (III) *via* Br⋯Br and Br⋯O contacts.

Quite different to the packing in (I) and (II), the mol­ecules in (III) are connected *via* pairs of C3—H3⋯F1 inter­actions (Fig. 7 ▸ and Table 3 ▸), forming 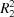
(8) dimers. Further, these dimers are connected through Br1⋯Br2 contacts [3.5253 (1) Å] along the *b* axis, forming ribbons. These ribbons are further inter­linked into columns *via* C8—Br2⋯O1=C7 contacts (Table 6 ▸), forming a two-dimensional architecture (Fig. 8 ▸). The packing in (III) does not features conventional N—H⋯O hydrogen bonds, similar to (I).

## Database survey   

Seven *N*-ar­yl-2,2,2-tri­bromo­acetamides, namely, 2,2,2-tri­bromo-*N*-phen­yl­acetamide, 2,2,2-tri­bromo-*N*-(2/3/4-chloro­phen­yl)­acetamides and 2,2,2-tri­bromo-*N*-(2/3/4-methyl­phen­yl)­acetamides have been previously reported. Comparison of the crystal systems of these series of compounds show that all the chloro-substituted compounds crystallize in the ortho­rhom­bic crystal system, while the methyl-substituted compounds crystallize in the monoclinic system (Table 7 ▸). However, such trends are not observed in fluoro-substituted compounds *i.e.* (I) and (III). Further, the asymmetric units of the fluoro- and chloro-substituted compounds contain one mol­ecule, whereas the asymmetric units of the methyl-substituted tri­bromo­acetamides contain two mol­ecules.

In (I), the conformation of the N—H bond is *syn* to the 2-fluoro substituent in the benzene ring, similar to that observed in the crystal structures of 2,2,2-tri­bromo-*N*-(2-chloro­phen­yl)­acetamide (I*a*) (Gowda *et al.*, 2010*a* ▸) and 2,2,2-tri­bromo-*N*-(2-methyl­phen­yl)­acetamide (I*b*) (Gowda *et al.*, 2010*b* ▸). In contrast to the above, in (II) the conformation of the N—H bond is *anti* to the 3-CF_3_ substituent, as observed in the other *meta-*substituted compounds *i.e.* 2,2,2-tri­bromo-*N*-(3-chloro­phen­yl)­acetamide (I*a*) (Suchetan *et al.*, 2010 ▸) and 2,2,2-tri­bromo-*N*-(3-methyl­phen­yl)­acetamide (I*b*) (Gowda *et al.*, 2009*c* ▸). Further, it can be observed that the mol­ecular structure of each of the compounds features intra­molecular N—H⋯Br hydrogen bonds, while the 2-fluoro and 2-chloro derivatives feature additional N—H⋯*X* (*X* = F or Cl) intra­molecular hydrogen bonds. Further, compounds (I), (II) and (III) exhibit C—H⋯O intra­molecular hydrogen bonds which are not displayed in the structures reported in the literature.

A comparison of the dihedral angle between the benzene ring and the C1–N1–C7(O)–C8 segment in all of the compounds shows that the dihedral angles in the fluoro-substituted compounds are smaller than those observed in chloro-substituted ones, which in turn have smaller values than the methyl-substituted tri­bromo­acetamides (Table 7 ▸). The dihedral angle in the parent (*i.e.* unsubstituted) compound is closer to those of chloro-substituted ones, thus the order is F < Cl(=H) < CH_3_.

The crystal structures of all of the seven compounds [except (I*a*)] reported in the literature feature strong N—H⋯O hydrogen bonds leading into *C*(4) chains forming a one-dimensional architecture. Compound (I*a*) (2-chloro derivative) does not exhibit any conventional inter­molecular inter­actions and therefore exhibits a zero-dimensional supra­molecular architecture. However, the packing of mol­ecules in the three structures reported here are very different and are controlled by inter­actions mainly involving the halogen atoms.

## Synthesis and crystallization   

All three compounds were prepared according to a literature method (Gowda *et al.*, 2003 ▸). The purity of the compounds was checked by determining the melting points. Single crystals of all the compounds used for X-ray diffraction studies were obtained by slow evaporation of an ethano­lic solutions of the compound at room temperature.

## Refinement   

Crystal data, data collection and structure refinement details are summarized in Table 8 ▸. H atoms were placed in geometrically idealized positions and constrained to ride on their parent atoms, with C—H = 0.93 Å and N—H = 0.86 Å, and with *U*_iso_(H) = 1.2*U*_eq_(C,N).

## Supplementary Material

Crystal structure: contains datablock(s) I, II, III, global. DOI: 10.1107/S2056989015015248/hb7472sup1.cif

Structure factors: contains datablock(s) I. DOI: 10.1107/S2056989015015248/hb7472Isup2.hkl

Structure factors: contains datablock(s) II. DOI: 10.1107/S2056989015015248/hb7472IIsup3.hkl

Structure factors: contains datablock(s) III. DOI: 10.1107/S2056989015015248/hb7472IIIsup4.hkl

CCDC references: 1064065, 1419188, 1419189

Additional supporting information:  crystallographic information; 3D view; checkCIF report

## Figures and Tables

**Table 1:** Hydrogen-bond geometry (Å, °) for (I)

*D*—H⋯*A*	*D*—H	H⋯*A*	*D*⋯*A*	*D*—H⋯*A*
N1—H1⋯Br3	0.86	2.56	3.056 (4)	118
N1—H1⋯F1	0.86	2.26	2.646 (6)	107
C6—H6⋯O1	0.93	2.32	2.896 (7)	120

**Table 2:** Hydrogen-bond geometry (Å, °) for (II)

*D*—H⋯*A*	*D*—H	H⋯*A*	*D*⋯*A*	*D*—H⋯*A*
N1—H1⋯Br1	0.86	2.78	3.144 (4)	108
C2—H2⋯O2	0.93	2.34	2.893 (6)	118
N1—H1⋯O2^i^	0.86	2.24	3.072 (5)	161
C6—H6⋯O2^i^	0.93	2.58	3.357 (5)	142

Symmetry code: (i) 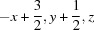.

**Table 3:** Hydrogen-bond geometry (Å, °) for (III)

*D*—H⋯*A*	*D*—H	H⋯*A*	*D*⋯*A*	*D*—H⋯*A*
N1—H1⋯Br1	0.86	2.49	3.051 (5)	124
C2—H2⋯O1	0.93	2.35	2.912 (8)	118
C3—H3⋯F1^i^	0.93	2.46	3.308 (8)	151

Symmetry code: (i) 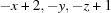.

**Table 4:** Halogen contacts in (I) *Cg* is the centroid of the C1–C6 aromatic ring.

C—*X*⋯*Y*	*X*⋯*Y*	C—*X*⋯*Y*
C8—Br2⋯*Cg* ^i^	3.426 (3)	174.52 (15)
C8—Br1⋯Br1^ii^	3.6519 (12)	141.04 (14)

Symmetry codes: (i) 2 − *x*, 1 − *y*, 1 − *z*; 2 − *x*, 2 − *y*, −*z*.

**Table 5:** Halogen contacts in (II) *Cg* is the centroid of the C1–C6 aromatic ring.

C—*X*⋯*Y*	*X*⋯*Y*	C—*X*⋯*Y*
C8—Br1⋯*Cg* ^i^	3.7543 (18)	119.96 (13)
C9—F2⋯*Cg* ^ii^	3.195 (4)	109.5 (3)
C8—Br1⋯Br3^iii^	3.6589 (6)	113.06 (2)
C8—Br2⋯F2^iv^	3.0290 (6)	1769.9 (2)

Symmetry codes: (i) 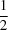 + *x*, *y*, 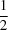 − *z*; (ii) −*x*, 1 − *y*, −*z*; (iii) 1 − *x*, −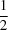 + *y*, 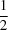 − *z*; (iv) 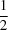 − *x*, −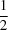 + *y*, *z*.

**Table 6:** Halogen contacts in (III)

C—*X*⋯*Y*	*X*⋯*Y*	C—*X*⋯*Y*
C8—Br2⋯Br1^i^	3.5254 (9)	158.87 (16)
C8—Br2⋯O1^ii^	3.0623 (4)	160.06 (18)

Symmetry codes: (i) *x*, 1 + *y*, *z*; *x*, −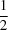 − *y*, 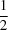 + *z*.

**Table 7:** Comparison of various parameters in the crystal structures of *N*-(ar­yl)-2,2,2-tri­bromo­acetamides

Parameters	H	2-F	2-Cl	2-CH_3_	3-CF_3_	3-Cl	3-CH_3_	4-F	4-Cl	4-CH_3_
Crystal system	ortho­rhom­bic	triclinic	ortho­rhom­bic	monoclinic	ortho­rhom­bic	ortho­rhom­bic	monoclinic	monoclinic	ortho­rhom­bic	monoclinic
*Z*′	1	1	1	2	1	1	2	1	1	2
Intra­molecular hydrogen bonds	N—H⋯Br	N—H⋯Br, N—H⋯F, C—H⋯O	N—H⋯Br, N—H⋯Cl	N—H⋯Br	N—H⋯Br, C—H⋯O	N—H⋯Br	N—H⋯Br	N—H⋯Br, C—H⋯O	N—H⋯Br	N—H⋯Br
Orientation of the substituent to the N—H bond	-	*syn*	*syn*	*syn*	*anti*	*anti*	*anti*	-	-	-
Dihedral angle between the benzene ring and the central chain	38.1 (10)	4.2 (3)	40.5 (3)	67.7 (5), 87.2 (5)	19.29 (1)	32.0 (6)	36.2 (5), 52.9 (6)	22.5 (3)	35.1 (5)	22.5 (5), 48.4 (5)
Inter­molecular inter­actions	N—H⋯O	Br⋯Br, C—Br⋯π	-	N—H⋯O	N—H⋯O, C—H⋯O, Br⋯Br, Br⋯F, C—Br⋯π, C—F⋯π	N—H⋯O	N—H⋯O	C—H⋯F, Br⋯Br, Br⋯O	N—H⋯O	N—H⋯O
Supra­molecular architecture	1D chains	1D chains	0D	1D chains	2D	1D chains	1D chains	2D	1D chains	1D chains

**Table 8:** Experimental details

	(I)	(II)	(III)
Crystal data			
Chemical formula	C_8_H_5_Br_3_FNO	C_9_H_5_Br_3_F_3_NO	C_8_H_5_Br_3_FNO
*M*_r_	389.86	439.87	389.86
Crystal system, space group	Triclinic, *P* 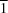	Orthorhombic, *P* *b* *c* *a*	Monoclinic, *P*2_1_/*c*
Temperature (K)	296	100	100
*a*, *b*, *c* (Å)	6.1825 (13), 8.929 (2), 9.971 (2)	11.3441 (6), 10.3047 (6), 20.6397 (11)	16.9830 (9), 6.1095 (3), 10.1508 (6)
α, β, γ (°)	85.858 (8), 87.966 (8), 78.919 (8)	90, 90, 90	90, 100.485 (1), 90
*V* (Å^3^)	538.6 (2)	2412.7 (2)	1035.64 (10)
*Z*	2	8	4
Radiation type	Cu *K*α	Cu *K*α	Cu *K*α
μ (mm^−1^)	13.77	12.66	14.33
Crystal size (mm)	0.28 × 0.24 × 0.22	0.30 × 0.27 × 0.25	0.31 × 0.26 × 0.22

Data collection			
Diffractometer	Bruker APEXII	Bruker APEXII	Bruker APEXII
Absorption correction	Multi-scan (*SADABS*; Bruker, 2009 ▸)	Multi-scan (*SADABS*; Bruker, 2009 ▸)	Multi-scan (*SADABS*; Bruker, 2009 ▸)
*T*_min_, *T*_max_	0.048, 0.053	0.116, 0.144	0.029, 0.043
No. of measured, independent and observed [*I* > 2σ(*I*)] reflections	4683, 1549, 1485	11524, 1978, 1967	6934, 1674, 1664
*R*_int_	0.051	0.054	0.054
θ_max_ (°)	60.0	64.5	64.3
(sin θ/λ)_max_ (Å^−1^)	0.562	0.585	0.584

Refinement			
*R*[*F* ^2^ > 2σ(*F* ^2^)], *wR*(*F* ^2^), *S*	0.045, 0.120, 1.10	0.040, 0.102, 1.22	0.047, 0.128, 1.19
No. of reflections	1549	1978	1674
No. of parameters	128	154	127
H-atom treatment	H-atom parameters constrained	H-atom parameters constrained	H-atom parameters constrained
Δρ_max_, Δρ_min_ (e Å^−3^)	0.96, −0.60	0.96, −0.81	1.48, −1.01

Computer programs: *APEX2*, *SAINT-Plus* and *XPREP* (Bruker, 2009 ▸), *SHELXS97* and *SHELXL97* (Sheldrick, 2008 ▸) and *Mercury* (Macrae *et al.*, 2008 ▸).

## supplementary crystallographic information

### (I) 2,2,2-Tribromo-N-(2-fluorophenyl)acetamide Crystal data

**Table:**

C_8_H_5_Br_3_FNO	*F*(000) = 364
*M**_r_* = 389.86	Prism
Triclinic, *P*1	*D*_x_ = 2.404 Mg m^−^^3^
Hall symbol: -P 1	Melting point: 403 K
*a* = 6.1825 (13) Å	Cu *K*α radiation, λ = 1.54178 Å
*b* = 8.929 (2) Å	Cell parameters from 123 reflections
*c* = 9.971 (2) Å	θ = 7.3–60.0°
α = 85.858 (8)°	µ = 13.77 mm^−^^1^
β = 87.966 (8)°	*T* = 296 K
γ = 78.919 (8)°	Prism, colourless
*V* = 538.6 (2) Å^3^	0.28 × 0.24 × 0.22 mm
*Z* = 2	

### (I) 2,2,2-Tribromo-N-(2-fluorophenyl)acetamide Data collection

**Table:**

Bruker APEXII diffractometer	1485 reflections with *I* > 2σ(*I*)
Radiation source: fine-focus sealed tube	*R*_int_ = 0.051
Graphite monochromator	θ_max_ = 60.0°, θ_min_ = 7.3°
phi and φ scans	*h* = −6→6
Absorption correction: multi-scan (SADABS; Bruker, 2009)	*k* = −10→10
*T*_min_ = 0.048, *T*_max_ = 0.053	*l* = −11→10
4683 measured reflections	1 standard reflections every 1 reflections
1549 independent reflections	intensity decay: 0.1%

### (I) 2,2,2-Tribromo-N-(2-fluorophenyl)acetamide Refinement

**Table:**

Refinement on *F*^2^	Secondary atom site location: difference Fourier map
Least-squares matrix: full	Hydrogen site location: inferred from neighbouring sites
*R*[*F*^2^ > 2σ(*F*^2^)] = 0.045	H-atom parameters constrained
*wR*(*F*^2^) = 0.120	*w* = 1/[σ^2^(*F*_o_^2^) + (0.073*P*)^2^ + 0.4735*P*] where *P* = (*F*_o_^2^ + 2*F*_c_^2^)/3
*S* = 1.10	(Δ/σ)_max_ < 0.001
1549 reflections	Δρ_max_ = 0.96 e Å^−^^3^
128 parameters	Δρ_min_ = −0.60 e Å^−^^3^
0 restraints	Extinction correction: SHELXL, Fc^*^=kFc[1+0.001xFc^2^λ^3^/sin(2θ)]^-1/4^
Primary atom site location: structure-invariant direct methods	Extinction coefficient: 0.0074 (12)

### (I) 2,2,2-Tribromo-N-(2-fluorophenyl)acetamide Special details

**Table:**

Geometry. All s.u.'s (except the s.u. in the dihedral angle between two l.s. planes) are estimated using the full covariance matrix. The cell s.u.'s are taken into account individually in the estimation of s.u.'s in distances, angles and torsion angles; correlations between s.u.'s in cell parameters are only used when they are defined by crystal symmetry. An approximate (isotropic) treatment of cell s.u.'s is used for estimating s.u.'s involving l.s. planes.
Refinement. Refinement of *F*^2^ against ALL reflections. The weighted *R*-factor *wR* and goodness of fit *S* are based on *F*^2^, conventional *R*-factors *R* are based on *F*, with *F* set to zero for negative *F*^2^. The threshold expression of *F*^2^ > 2σ(*F*^2^) is used only for calculating *R*-factors(gt) *etc*. and is not relevant to the choice of reflections for refinement. *R*-factors based on *F*^2^ are statistically about twice as large as those based on *F*, and *R*- factors based on ALL data will be even larger.

### (I) 2,2,2-Tribromo-N-(2-fluorophenyl)acetamide Fractional atomic coordinates and isotropic or equivalent isotropic displacement parameters (Å^2^)

**Table:**

	*x*	*y*	*z*	*U*_iso_*/*U*_eq_	
F1	0.3498 (7)	0.6456 (4)	0.5450 (4)	0.0760 (11)	
C2	0.4528 (10)	0.7432 (6)	0.6065 (5)	0.0501 (12)	
C1	0.6308 (9)	0.7870 (5)	0.5395 (5)	0.0429 (11)	
C6	0.7390 (10)	0.8852 (6)	0.6027 (5)	0.0536 (13)	
H6	0.8603	0.9181	0.5613	0.064*	
C5	0.6636 (11)	0.9331 (6)	0.7277 (5)	0.0569 (14)	
H5	0.7358	0.9984	0.7698	0.068*	
C4	0.4852 (11)	0.8866 (7)	0.7908 (6)	0.0607 (14)	
H4	0.4378	0.9204	0.8748	0.073*	
C3	0.3764 (11)	0.7905 (7)	0.7305 (6)	0.0614 (15)	
H3	0.2547	0.7583	0.7722	0.074*	
N1	0.6900 (8)	0.7303 (5)	0.4129 (4)	0.0487 (10)	
H1	0.6157	0.6658	0.3871	0.058*	
C7	0.8475 (8)	0.7644 (5)	0.3276 (5)	0.0430 (11)	
O1	0.9690 (8)	0.8512 (5)	0.3453 (4)	0.0735 (14)	
C8	0.8737 (8)	0.6801 (5)	0.1956 (5)	0.0410 (11)	
Br1	0.99546 (11)	0.80114 (7)	0.05528 (5)	0.0602 (3)	
Br2	1.07967 (10)	0.48905 (6)	0.23436 (7)	0.0669 (3)	
Br3	0.60118 (10)	0.63535 (7)	0.13471 (6)	0.0600 (3)	

### (I) 2,2,2-Tribromo-N-(2-fluorophenyl)acetamide Atomic displacement parameters (Å^2^)

**Table:**

	*U*^11^	*U*^22^	*U*^33^	*U*^12^	*U*^13^	*U*^23^
F1	0.073 (2)	0.089 (2)	0.081 (2)	−0.049 (2)	0.0223 (19)	−0.0244 (19)
C2	0.054 (3)	0.045 (3)	0.055 (3)	−0.019 (2)	0.003 (2)	−0.003 (2)
C1	0.047 (3)	0.041 (2)	0.040 (2)	−0.010 (2)	0.001 (2)	0.0047 (19)
C6	0.065 (4)	0.053 (3)	0.047 (3)	−0.023 (3)	0.004 (2)	0.001 (2)
C5	0.070 (4)	0.053 (3)	0.050 (3)	−0.018 (3)	−0.008 (3)	−0.005 (2)
C4	0.073 (4)	0.057 (3)	0.051 (3)	−0.011 (3)	0.009 (3)	−0.005 (2)
C3	0.063 (4)	0.058 (3)	0.061 (3)	−0.012 (3)	0.023 (3)	−0.003 (3)
N1	0.057 (3)	0.048 (2)	0.048 (2)	−0.026 (2)	0.009 (2)	−0.0048 (17)
C7	0.041 (3)	0.042 (3)	0.046 (2)	−0.012 (2)	0.000 (2)	0.0031 (19)
O1	0.078 (3)	0.096 (3)	0.064 (2)	−0.060 (3)	0.017 (2)	−0.022 (2)
C8	0.033 (2)	0.046 (3)	0.046 (2)	−0.0129 (19)	0.0032 (19)	−0.001 (2)
Br1	0.0689 (5)	0.0687 (5)	0.0477 (4)	−0.0297 (3)	0.0123 (3)	0.0032 (3)
Br2	0.0537 (5)	0.0484 (5)	0.0950 (6)	−0.0024 (3)	−0.0021 (3)	−0.0005 (3)
Br3	0.0455 (5)	0.0839 (5)	0.0573 (5)	−0.0263 (3)	−0.0035 (3)	−0.0095 (3)

### (I) 2,2,2-Tribromo-N-(2-fluorophenyl)acetamide Geometric parameters (Å, º)

**Table:**

F1—C2	1.363 (6)	C4—H4	0.9300
C2—C3	1.374 (8)	C3—H3	0.9300
C2—C1	1.373 (8)	N1—C7	1.334 (6)
C1—C6	1.396 (7)	N1—H1	0.8600
C1—N1	1.405 (6)	C7—O1	1.204 (6)
C6—C5	1.382 (8)	C7—C8	1.551 (7)
C6—H6	0.9300	C8—Br1	1.927 (4)
C5—C4	1.369 (9)	C8—Br3	1.932 (5)
C5—H5	0.9300	C8—Br2	1.946 (5)
C4—C3	1.370 (9)		
			
F1—C2—C3	119.2 (5)	C2—C3—C4	117.8 (6)
F1—C2—C1	116.9 (5)	C2—C3—H3	121.1
C3—C2—C1	123.9 (5)	C4—C3—H3	121.1
C2—C1—C6	117.3 (5)	C7—N1—C1	127.9 (4)
C2—C1—N1	117.8 (5)	C7—N1—H1	116.1
C6—C1—N1	124.9 (5)	C1—N1—H1	116.1
C5—C6—C1	119.2 (5)	O1—C7—N1	126.1 (5)
C5—C6—H6	120.4	O1—C7—C8	118.7 (4)
C1—C6—H6	120.4	N1—C7—C8	115.3 (4)
C4—C5—C6	121.5 (5)	C7—C8—Br1	109.6 (3)
C4—C5—H5	119.2	C7—C8—Br3	113.7 (3)
C6—C5—H5	119.2	Br1—C8—Br3	108.8 (2)
C5—C4—C3	120.2 (5)	C7—C8—Br2	105.9 (3)
C5—C4—H4	119.9	Br1—C8—Br2	109.6 (2)
C3—C4—H4	119.9	Br3—C8—Br2	109.1 (2)
			
F1—C2—C1—C6	−179.1 (5)	C2—C1—N1—C7	175.8 (5)
C3—C2—C1—C6	0.1 (8)	C6—C1—N1—C7	−4.0 (8)
F1—C2—C1—N1	1.0 (7)	C1—N1—C7—O1	0.2 (9)
C3—C2—C1—N1	−179.7 (5)	C1—N1—C7—C8	179.3 (5)
C2—C1—C6—C5	0.0 (8)	O1—C7—C8—Br1	−27.4 (6)
N1—C1—C6—C5	179.8 (5)	N1—C7—C8—Br1	153.5 (4)
C1—C6—C5—C4	−0.1 (9)	O1—C7—C8—Br3	−149.4 (5)
C6—C5—C4—C3	0.0 (9)	N1—C7—C8—Br3	31.5 (5)
F1—C2—C3—C4	179.0 (6)	O1—C7—C8—Br2	90.8 (5)
C1—C2—C3—C4	−0.2 (9)	N1—C7—C8—Br2	−88.3 (4)
C5—C4—C3—C2	0.2 (9)		

### (I) 2,2,2-Tribromo-N-(2-fluorophenyl)acetamide Hydrogen-bond geometry (Å, º)

**Table:**

*D*—H···*A*	*D*—H	H···*A*	*D*···*A*	*D*—H···*A*
N1—H1···Br3	0.86	2.56	3.056 (4)	118
N1—H1···F1	0.86	2.26	2.646 (6)	107
C6—H6···O1	0.93	2.32	2.896 (7)	120

### (II) 2,2,2-Tribromo-N-[3-(trifluoromethyl)phenyl]acetamide Crystal data

**Table:**

C_9_H_5_Br_3_F_3_NO	Prism
*M**_r_* = 439.87	*D*_x_ = 2.422 Mg m^−^^3^
Orthorhombic, *P**b**c**a*	Melting point: 425 K
Hall symbol: -P 2ac 2ab	Cu *K*α radiation, λ = 1.54178 Å
*a* = 11.3441 (6) Å	Cell parameters from 145 reflections
*b* = 10.3047 (6) Å	θ = 5.8–64.5°
*c* = 20.6397 (11) Å	µ = 12.66 mm^−^^1^
*V* = 2412.7 (2) Å^3^	*T* = 100 K
*Z* = 8	Prism, colourless
*F*(000) = 1648	0.30 × 0.27 × 0.25 mm

### (II) 2,2,2-Tribromo-N-[3-(trifluoromethyl)phenyl]acetamide Data collection

**Table:**

Bruker APEXII diffractometer	1967 reflections with *I* > 2σ(*I*)
Radiation source: fine-focus sealed tube	*R*_int_ = 0.054
Graphite monochromator	θ_max_ = 64.5°, θ_min_ = 5.8°
phi and φ scans	*h* = −13→13
Absorption correction: multi-scan (SADABS; Bruker, 2009)	*k* = −11→6
*T*_min_ = 0.116, *T*_max_ = 0.144	*l* = −23→24
11524 measured reflections	1 standard reflections every 1 reflections
1978 independent reflections	intensity decay: 0.1%

### (II) 2,2,2-Tribromo-N-[3-(trifluoromethyl)phenyl]acetamide Refinement

**Table:**

Refinement on *F*^2^	Primary atom site location: structure-invariant direct methods
Least-squares matrix: full	Secondary atom site location: difference Fourier map
*R*[*F*^2^ > 2σ(*F*^2^)] = 0.040	Hydrogen site location: inferred from neighbouring sites
*wR*(*F*^2^) = 0.102	H-atom parameters constrained
*S* = 1.22	*w* = 1/[σ^2^(*F*_o_^2^) + (0.0569*P*)^2^ + 5.8187*P*] where *P* = (*F*_o_^2^ + 2*F*_c_^2^)/3
1978 reflections	(Δ/σ)_max_ = 0.001
154 parameters	Δρ_max_ = 0.96 e Å^−^^3^
0 restraints	Δρ_min_ = −0.81 e Å^−^^3^

### (II) 2,2,2-Tribromo-N-[3-(trifluoromethyl)phenyl]acetamide Special details

**Table:**

Geometry. All s.u.'s (except the s.u. in the dihedral angle between two l.s. planes) are estimated using the full covariance matrix. The cell s.u.'s are taken into account individually in the estimation of s.u.'s in distances, angles and torsion angles; correlations between s.u.'s in cell parameters are only used when they are defined by crystal symmetry. An approximate (isotropic) treatment of cell s.u.'s is used for estimating s.u.'s involving l.s. planes.
Refinement. Refinement of *F*^2^ against ALL reflections. The weighted *R*-factor *wR* and goodness of fit *S* are based on *F*^2^, conventional *R*-factors *R* are based on *F*, with *F* set to zero for negative *F*^2^. The threshold expression of *F*^2^ > 2σ(*F*^2^) is used only for calculating *R*-factors(gt) *etc*. and is not relevant to the choice of reflections for refinement. *R*-factors based on *F*^2^ are statistically about twice as large as those based on *F*, and *R*- factors based on ALL data will be even larger.

### (II) 2,2,2-Tribromo-N-[3-(trifluoromethyl)phenyl]acetamide Fractional atomic coordinates and isotropic or equivalent isotropic displacement parameters (Å^2^)

**Table:**

	*x*	*y*	*z*	*U*_iso_*/*U*_eq_	
Br1	0.82269 (4)	0.22572 (4)	0.72340 (2)	0.01621 (19)	
Br2	0.97513 (4)	0.19720 (5)	0.85195 (2)	0.0197 (2)	
Br3	0.93377 (4)	−0.04340 (4)	0.76175 (2)	0.0208 (2)	
F1	0.2435 (3)	−0.0016 (4)	0.89656 (15)	0.0418 (9)	
F2	0.3744 (3)	−0.1282 (3)	0.93617 (17)	0.0340 (8)	
F3	0.2658 (2)	−0.0138 (3)	0.99878 (14)	0.0237 (6)	
N1	0.6923 (3)	0.1851 (4)	0.85629 (17)	0.0120 (8)	
H1	0.7205	0.2589	0.8445	0.014*	
O2	0.7243 (3)	−0.0319 (3)	0.84716 (15)	0.0149 (7)	
C4	0.3873 (4)	0.2127 (4)	0.9709 (2)	0.0172 (10)	
H4	0.3194	0.2210	0.9959	0.021*	
C3	0.4123 (4)	0.0974 (4)	0.9396 (2)	0.0131 (9)	
C2	0.5142 (4)	0.0835 (4)	0.9018 (2)	0.0118 (8)	
H2	0.5306	0.0054	0.8811	0.014*	
C1	0.5903 (4)	0.1887 (4)	0.8958 (2)	0.0113 (8)	
C7	0.7500 (4)	0.0795 (4)	0.83513 (19)	0.0102 (8)	
C8	0.8624 (4)	0.1125 (4)	0.7951 (2)	0.0120 (8)	
C9	0.3251 (4)	−0.0103 (5)	0.9422 (2)	0.0160 (9)	
C5	0.4656 (4)	0.3166 (5)	0.9645 (2)	0.0169 (9)	
H5	0.4501	0.3945	0.9856	0.020*	
C6	0.5654 (4)	0.3043 (4)	0.9272 (2)	0.0150 (9)	
H6	0.6167	0.3742	0.9231	0.018*	

### (II) 2,2,2-Tribromo-N-[3-(trifluoromethyl)phenyl]acetamide Atomic displacement parameters (Å^2^)

**Table:**

	*U*^11^	*U*^22^	*U*^33^	*U*^12^	*U*^13^	*U*^23^
Br1	0.0198 (3)	0.0158 (3)	0.0130 (3)	0.00137 (17)	0.00429 (17)	0.00239 (17)
Br2	0.0118 (3)	0.0246 (3)	0.0226 (3)	−0.00234 (18)	−0.00244 (17)	−0.00310 (19)
Br3	0.0237 (3)	0.0121 (3)	0.0265 (3)	0.00441 (18)	0.0140 (2)	−0.00061 (18)
F1	0.0314 (17)	0.062 (2)	0.0323 (17)	−0.0301 (16)	−0.0199 (14)	0.0239 (17)
F2	0.0258 (16)	0.0208 (16)	0.055 (2)	−0.0061 (12)	0.0192 (14)	−0.0077 (14)
F3	0.0226 (14)	0.0253 (15)	0.0230 (14)	−0.0085 (12)	0.0105 (12)	−0.0016 (11)
N1	0.0119 (18)	0.0095 (18)	0.0147 (18)	−0.0009 (14)	0.0072 (14)	−0.0002 (14)
O2	0.0131 (16)	0.0123 (17)	0.0194 (15)	−0.0004 (12)	0.0038 (12)	0.0026 (12)
C4	0.015 (2)	0.019 (2)	0.018 (2)	0.0045 (18)	0.0053 (18)	−0.0009 (18)
C3	0.011 (2)	0.015 (2)	0.014 (2)	0.0036 (17)	−0.0014 (16)	0.0017 (17)
C2	0.011 (2)	0.012 (2)	0.013 (2)	0.0016 (16)	−0.0032 (16)	0.0004 (17)
C1	0.0088 (19)	0.013 (2)	0.012 (2)	0.0034 (16)	−0.0024 (17)	0.0012 (16)
C7	0.010 (2)	0.010 (2)	0.0104 (19)	−0.0016 (16)	−0.0014 (16)	−0.0004 (16)
C8	0.013 (2)	0.009 (2)	0.0146 (19)	0.0019 (17)	0.0038 (17)	0.0000 (17)
C9	0.015 (2)	0.019 (2)	0.015 (2)	0.0023 (18)	0.0028 (17)	−0.0012 (18)
C5	0.013 (2)	0.015 (2)	0.023 (2)	0.0031 (18)	0.0032 (18)	−0.0044 (19)
C6	0.013 (2)	0.012 (2)	0.020 (2)	0.0017 (16)	0.0001 (18)	0.0004 (18)

### (II) 2,2,2-Tribromo-N-[3-(trifluoromethyl)phenyl]acetamide Geometric parameters (Å, º)

**Table:**

Br1—C8	1.938 (4)	C4—C5	1.397 (7)
Br2—C8	1.943 (4)	C4—H4	0.9300
Br3—C8	1.926 (4)	C3—C2	1.402 (6)
F1—C9	1.324 (5)	C3—C9	1.488 (7)
F2—C9	1.343 (6)	C2—C1	1.391 (6)
F3—C9	1.348 (5)	C2—H2	0.9300
N1—C7	1.343 (6)	C1—C6	1.386 (6)
N1—C1	1.416 (6)	C7—C8	1.557 (6)
N1—H1	0.8600	C5—C6	1.376 (7)
O2—C7	1.211 (5)	C5—H5	0.9300
C4—C3	1.383 (7)	C6—H6	0.9300
			
C7—N1—C1	127.3 (4)	C7—C8—Br3	110.6 (3)
C7—N1—H1	116.3	C7—C8—Br1	110.2 (3)
C1—N1—H1	116.3	Br3—C8—Br1	109.1 (2)
C3—C4—C5	118.9 (4)	C7—C8—Br2	108.5 (3)
C3—C4—H4	120.5	Br3—C8—Br2	108.3 (2)
C5—C4—H4	120.5	Br1—C8—Br2	110.1 (2)
C4—C3—C2	121.2 (4)	F1—C9—F2	106.6 (4)
C4—C3—C9	119.2 (4)	F1—C9—F3	105.6 (3)
C2—C3—C9	119.5 (4)	F2—C9—F3	105.3 (4)
C1—C2—C3	118.8 (4)	F1—C9—C3	112.8 (4)
C1—C2—H2	120.6	F2—C9—C3	113.2 (4)
C3—C2—H2	120.6	F3—C9—C3	112.5 (4)
C6—C1—C2	120.1 (4)	C6—C5—C4	120.4 (4)
C6—C1—N1	117.3 (4)	C6—C5—H5	119.8
C2—C1—N1	122.6 (4)	C4—C5—H5	119.8
O2—C7—N1	125.8 (4)	C5—C6—C1	120.6 (4)
O2—C7—C8	120.8 (4)	C5—C6—H6	119.7
N1—C7—C8	113.3 (4)	C1—C6—H6	119.7
			
C5—C4—C3—C2	−0.1 (7)	N1—C7—C8—Br1	−55.4 (4)
C5—C4—C3—C9	−175.5 (4)	O2—C7—C8—Br2	−111.5 (4)
C4—C3—C2—C1	−0.4 (6)	N1—C7—C8—Br2	65.2 (4)
C9—C3—C2—C1	175.1 (4)	C4—C3—C9—F1	85.9 (5)
C3—C2—C1—C6	0.5 (6)	C2—C3—C9—F1	−89.7 (5)
C3—C2—C1—N1	−177.1 (4)	C4—C3—C9—F2	−152.9 (4)
C7—N1—C1—C6	161.6 (4)	C2—C3—C9—F2	31.6 (6)
C7—N1—C1—C2	−20.8 (7)	C4—C3—C9—F3	−33.6 (6)
C1—N1—C7—O2	−0.8 (7)	C2—C3—C9—F3	150.9 (4)
C1—N1—C7—C8	−177.3 (4)	C3—C4—C5—C6	0.5 (7)
O2—C7—C8—Br3	7.2 (5)	C4—C5—C6—C1	−0.4 (7)
N1—C7—C8—Br3	−176.1 (3)	C2—C1—C6—C5	−0.1 (7)
O2—C7—C8—Br1	127.9 (4)	N1—C1—C6—C5	177.6 (4)

### (II) 2,2,2-Tribromo-N-[3-(trifluoromethyl)phenyl]acetamide Hydrogen-bond geometry (Å, º)

**Table:**

*D*—H···*A*	*D*—H	H···*A*	*D*···*A*	*D*—H···*A*
N1—H1···Br1	0.86	2.78	3.144 (4)	108
C2—H2···O2	0.93	2.34	2.893 (6)	118
N1—H1···O2^i^	0.86	2.24	3.072 (5)	161
C6—H6···O2^i^	0.93	2.58	3.357 (5)	142

Symmetry code: (i) −*x*+3/2, *y*+1/2, *z*.

### (III) 2,2,2-Tribromo-N-(4-fluorophenyl)acetamide Crystal data

**Table:**

C_8_H_5_Br_3_FNO	Prism
*M**_r_* = 389.86	*D*_x_ = 2.500 Mg m^−^^3^
Monoclinic, *P*2_1_/*c*	Melting point: 434 K
Hall symbol: -P 2ybc	Cu *K*α radiation, λ = 1.54178 Å
*a* = 16.9830 (9) Å	Cell parameters from 133 reflections
*b* = 6.1095 (3) Å	θ = 5.3–64.3°
*c* = 10.1508 (6) Å	µ = 14.33 mm^−^^1^
β = 100.485 (1)°	*T* = 100 K
*V* = 1035.64 (10) Å^3^	Prism, colourless
*Z* = 4	0.31 × 0.26 × 0.22 mm
*F*(000) = 728	

### (III) 2,2,2-Tribromo-N-(4-fluorophenyl)acetamide Data collection

**Table:**

Bruker APEXII diffractometer	1664 reflections with *I* > 2σ(*I*)
Radiation source: fine-focus sealed tube	*R*_int_ = 0.054
Graphite monochromator	θ_max_ = 64.3°, θ_min_ = 5.3°
phi and φ scans	*h* = −19→19
Absorption correction: multi-scan (SADABS; Bruker, 2009)	*k* = −4→7
*T*_min_ = 0.029, *T*_max_ = 0.043	*l* = −11→11
6934 measured reflections	1 standard reflections every 1 reflections
1674 independent reflections	intensity decay: 0.1%

### (III) 2,2,2-Tribromo-N-(4-fluorophenyl)acetamide Refinement

**Table:**

Refinement on *F*^2^	Primary atom site location: structure-invariant direct methods
Least-squares matrix: full	Secondary atom site location: difference Fourier map
*R*[*F*^2^ > 2σ(*F*^2^)] = 0.047	Hydrogen site location: inferred from neighbouring sites
*wR*(*F*^2^) = 0.128	H-atom parameters constrained
*S* = 1.19	*w* = 1/[σ^2^(*F*_o_^2^) + (0.0755*P*)^2^ + 4.744*P*] where *P* = (*F*_o_^2^ + 2*F*_c_^2^)/3
1674 reflections	(Δ/σ)_max_ = 0.001
127 parameters	Δρ_max_ = 1.48 e Å^−^^3^
0 restraints	Δρ_min_ = −1.01 e Å^−^^3^

### (III) 2,2,2-Tribromo-N-(4-fluorophenyl)acetamide Special details

**Table:**

Geometry. All s.u.'s (except the s.u. in the dihedral angle between two l.s. planes) are estimated using the full covariance matrix. The cell s.u.'s are taken into account individually in the estimation of s.u.'s in distances, angles and torsion angles; correlations between s.u.'s in cell parameters are only used when they are defined by crystal symmetry. An approximate (isotropic) treatment of cell s.u.'s is used for estimating s.u.'s involving l.s. planes.
Refinement. Refinement of *F*^2^ against ALL reflections. The weighted *R*-factor *wR* and goodness of fit *S* are based on *F*^2^, conventional *R*-factors *R* are based on *F*, with *F* set to zero for negative *F*^2^. The threshold expression of *F*^2^ > 2σ(*F*^2^) is used only for calculating *R*-factors(gt) *etc*. and is not relevant to the choice of reflections for refinement. *R*-factors based on *F*^2^ are statistically about twice as large as those based on *F*, and *R*- factors based on ALL data will be even larger.

### (III) 2,2,2-Tribromo-N-(4-fluorophenyl)acetamide Fractional atomic coordinates and isotropic or equivalent isotropic displacement parameters (Å^2^)

**Table:**

	*x*	*y*	*z*	*U*_iso_*/*U*_eq_	
C1	0.8156 (3)	0.2042 (11)	0.7648 (6)	0.0142 (12)	
C2	0.8352 (4)	0.0287 (10)	0.6916 (6)	0.0152 (12)	
H2	0.8084	−0.1037	0.6933	0.018*	
C3	0.8953 (4)	0.0494 (11)	0.6151 (7)	0.0219 (14)	
H3	0.9094	−0.0684	0.5662	0.026*	
C4	0.9331 (4)	0.2482 (12)	0.6135 (6)	0.0218 (14)	
C5	0.9147 (4)	0.4249 (11)	0.6834 (7)	0.0199 (14)	
H5	0.9414	0.5570	0.6801	0.024*	
C6	0.8548 (4)	0.4041 (11)	0.7601 (6)	0.0174 (13)	
H6	0.8409	0.5233	0.8081	0.021*	
C7	0.7195 (3)	0.0190 (10)	0.8844 (6)	0.0127 (12)	
C8	0.6445 (3)	0.0612 (9)	0.9497 (6)	0.0116 (12)	
N1	0.7536 (3)	0.1979 (9)	0.8401 (5)	0.0138 (10)	
H1	0.7353	0.3224	0.8602	0.017*	
O1	0.7404 (3)	−0.1693 (7)	0.8719 (4)	0.0183 (9)	
F1	0.9926 (2)	0.2668 (7)	0.5404 (4)	0.0319 (10)	
Br1	0.62345 (4)	0.36497 (10)	0.98592 (6)	0.0175 (3)	
Br2	0.65806 (4)	−0.10117 (10)	1.11533 (6)	0.0141 (3)	
Br3	0.55374 (3)	−0.05639 (11)	0.82576 (6)	0.0182 (3)	

### (III) 2,2,2-Tribromo-N-(4-fluorophenyl)acetamide Atomic displacement parameters (Å^2^)

**Table:**

	*U*^11^	*U*^22^	*U*^33^	*U*^12^	*U*^13^	*U*^23^
C1	0.010 (3)	0.022 (3)	0.012 (3)	0.001 (2)	0.005 (2)	0.003 (3)
C2	0.014 (3)	0.011 (3)	0.022 (3)	0.001 (2)	0.009 (2)	0.003 (3)
C3	0.023 (3)	0.021 (4)	0.026 (3)	0.004 (3)	0.016 (3)	0.001 (3)
C4	0.015 (3)	0.028 (4)	0.027 (3)	0.007 (3)	0.014 (3)	0.008 (3)
C5	0.016 (3)	0.017 (3)	0.029 (4)	−0.003 (2)	0.008 (3)	0.006 (3)
C6	0.017 (3)	0.019 (3)	0.018 (3)	−0.002 (2)	0.007 (2)	−0.005 (2)
C7	0.010 (3)	0.016 (3)	0.014 (3)	0.000 (2)	0.008 (2)	0.001 (2)
C8	0.009 (3)	0.008 (3)	0.020 (3)	0.003 (2)	0.008 (2)	0.001 (2)
N1	0.014 (2)	0.010 (3)	0.021 (3)	0.0011 (19)	0.011 (2)	0.003 (2)
O1	0.022 (2)	0.008 (2)	0.029 (2)	0.0023 (17)	0.0151 (18)	−0.0017 (18)
F1	0.027 (2)	0.032 (2)	0.045 (2)	0.0015 (17)	0.0296 (18)	0.008 (2)
Br1	0.0218 (4)	0.0085 (4)	0.0261 (4)	0.0034 (2)	0.0147 (3)	0.0002 (2)
Br2	0.0184 (4)	0.0110 (4)	0.0144 (4)	0.0008 (2)	0.0073 (3)	0.0016 (2)
Br3	0.0118 (4)	0.0235 (4)	0.0193 (4)	0.0000 (2)	0.0027 (3)	−0.0035 (3)

### (III) 2,2,2-Tribromo-N-(4-fluorophenyl)acetamide Geometric parameters (Å, º)

**Table:**

C1—C2	1.379 (9)	C5—H5	0.9300
C1—C6	1.396 (9)	C6—H6	0.9300
C1—N1	1.410 (7)	C7—O1	1.218 (8)
C2—C3	1.397 (9)	C7—N1	1.352 (8)
C2—H2	0.9300	C7—C8	1.560 (7)
C3—C4	1.375 (10)	C8—Br2	1.929 (6)
C3—H3	0.9300	C8—Br1	1.938 (6)
C4—C5	1.359 (10)	C8—Br3	1.942 (6)
C4—F1	1.363 (7)	N1—H1	0.8600
C5—C6	1.395 (9)		
			
C2—C1—C6	119.9 (5)	C5—C6—C1	119.9 (6)
C2—C1—N1	123.3 (6)	C5—C6—H6	120.0
C6—C1—N1	116.7 (5)	C1—C6—H6	120.0
C1—C2—C3	120.1 (6)	O1—C7—N1	125.4 (5)
C1—C2—H2	119.9	O1—C7—C8	118.5 (5)
C3—C2—H2	119.9	N1—C7—C8	116.1 (5)
C4—C3—C2	118.4 (6)	C7—C8—Br2	107.9 (4)
C4—C3—H3	120.8	C7—C8—Br1	115.6 (4)
C2—C3—H3	120.8	Br2—C8—Br1	108.9 (3)
C5—C4—F1	118.8 (6)	C7—C8—Br3	106.1 (4)
C5—C4—C3	122.9 (6)	Br2—C8—Br3	109.2 (3)
F1—C4—C3	118.3 (6)	Br1—C8—Br3	109.0 (3)
C4—C5—C6	118.7 (6)	C7—N1—C1	127.6 (5)
C4—C5—H5	120.7	C7—N1—H1	116.2
C6—C5—H5	120.7	C1—N1—H1	116.2
			
C6—C1—C2—C3	1.3 (9)	O1—C7—C8—Br2	50.5 (6)
N1—C1—C2—C3	177.4 (6)	N1—C7—C8—Br2	−132.8 (4)
C1—C2—C3—C4	−0.8 (10)	O1—C7—C8—Br1	172.6 (4)
C2—C3—C4—C5	0.1 (10)	N1—C7—C8—Br1	−10.6 (7)
C2—C3—C4—F1	179.0 (6)	O1—C7—C8—Br3	−66.5 (6)
F1—C4—C5—C6	−178.8 (6)	N1—C7—C8—Br3	110.3 (5)
C3—C4—C5—C6	0.0 (10)	O1—C7—N1—C1	4.2 (9)
C4—C5—C6—C1	0.5 (10)	C8—C7—N1—C1	−172.4 (5)
C2—C1—C6—C5	−1.2 (9)	C2—C1—N1—C7	19.8 (9)
N1—C1—C6—C5	−177.5 (6)	C6—C1—N1—C7	−164.0 (6)

### (III) 2,2,2-Tribromo-N-(4-fluorophenyl)acetamide Hydrogen-bond geometry (Å, º)

**Table:**

*D*—H···*A*	*D*—H	H···*A*	*D*···*A*	*D*—H···*A*
N1—H1···Br1	0.86	2.49	3.051 (5)	124
C2—H2···O1	0.93	2.35	2.912 (8)	118
C3—H3···F1^i^	0.93	2.46	3.308 (8)	151

Symmetry code: (i) −*x*+2, −*y*, −*z*+1.
